# Rab25 acts as an oncogene in luminal B breast cancer and is causally associated with Snail driven EMT

**DOI:** 10.18632/oncotarget.9730

**Published:** 2016-05-31

**Authors:** Shreya Mitra, Lorenzo Federico, Wei Zhao, Jennifer Dennison, Tapasree Roy Sarkar, Fan Zhang, Vinita Takiar, Kwai W. Cheng, Sendurai Mani, Ju Seog Lee, Gordon B. Mills

**Affiliations:** ^1^ Department of Systems Biology, The University of Texas MD Anderson Cancer Center, Houston, TX, USA; ^2^ Center for Statistical Bioinformatics, Texas A&M University, College Station, TX, USA; ^3^ Department of Radiation Oncology, University of Cincinnati, Cincinnati, OH, USA; ^4^ Cardiac Catheterization Laboratory, Michael and DeBakey Medical Center, Houston, TX, USA; ^5^ Department of Translational Molecular Pathology, The University of Texas MD Anderson Cancer Center, Houston, TX, USA

**Keywords:** Rab25, Rab coupling protein, breast cancer, luminal B, claudin low

## Abstract

The Rab GTPases regulate vesicular trafficking machinery that transports and delivers a diverse pool of cargo, including growth factor receptors, integrins, nutrient receptors and junction proteins to specific intracellular sites. The trafficking machinery is indeed a major posttranslational modifier and is critical for cellular homeostasis. Deregulation of this stringently controlled system leads to a wide spectrum of disorders including cancer. Herein we demonstrate that Rab25, a key GTPase, mostly decorating the apical recycling endosome, is a dichotomous variable in breast cancer cell lines with higher mRNA and protein expression in Estrogen Receptor positive (ER+ve) lines. Rab25 and its effector, Rab Coupling Protein (RCP) are frequently coamplified and coordinately elevated in ER+ve breast cancers. In contrast, Rab25 levels are decreased in basal-like and almost completely lost in claudin-low tumors. This dichotomy exists despite the presence of the 1q amplicon that hosts Rab25 across breast cancer subtypes and is likely due to differential methylation of the Rab25 promoter. Functionally, elevated levels of Rab25 drive major hallmarks of cancer including indefinite growth and metastasis but in case of luminal B breast cancer only. Importantly, in such ER+ve tumors, coexpression of Rab25 and its effector, RCP is significantly associated with a markedly worsened clinical outcome. Importantly, in claudin-low cell lines, exogenous Rab25 markedly inhibits cell migration. Similarly, during Snail-induced epithelial to mesenchymal transition (EMT) exogenous Rab25 potently reverses Snail-driven invasion. Overall, this study substantiates a striking context dependent role of Rab25 in breast cancer where Rab25 is amplified and enhances aggressiveness in luminal B cancers while in claudin-low tumors, Rab25 is lost indicating possible anti-tumor functions.

## INTRODUCTION

The vesicular trafficking machinery regulated by Rab GTPase decorated vesicles [1] optimizes spatiotemporal delivery of extracellular and intracellular cargo to cellular hotspots by triggering downstream cellular signaling that drives growth, locomotion [2], development [3] and overall survival of organisms [4, 5]. Because the cargo [6] itself ranges from growth factors [7, 8], nutrient receptors [9], integrins [10–11], to junction proteins [12, 13], the potential of the trafficking machinery as a major post transcriptional regulator of protein function becomes evident, especially under limiting resource conditions. Cancer cells frequently co-opt and corrupt mechanisms used by normal cells to increase robustness or to mediate events associated with tumorigenesis and progression. With aberrations in this complex multicomponent resource management and transportation machinery leading to a multitude of inherited and acquired disorders including cancer [14], Rab GTPase family members are emerging as exploitable targets for disease therapy [15–17].

Rab25 (Catx-8, Rab11c), a member of the Rab family that plays a key role in governing apical and late endosomal recycling [18, 19] routes, is frequently amplified in epithelial cancers and is implicated in the pathophysiology of breast and ovarian [17, 20–22], cervical [23], head and neck [24], esophageal [25], gastric [26], colon [27], bladder [28] and kidney tumors [29–31]. We have demonstrated that the Rab25 amplicon and Rab25 expression is associated with a worsened outcome in ovarian cancer and increased the aggressiveness of a subset of breast cancer cell lines [32, 33]. Further, increased Rab25 is associated with poor patient outcome in lung [34], clear cell renal [35] and bladder [28] cancers likely through effects on metastatic pathways [36]. Mechanistically Rab25 decorated vesicles transport integrins [37, 38], PI3K [39], and EGFR [7] facilitating aggressive tumor growth and metastasis. Rab25 expression also correlates with cancer stem cell-like populations in a subset of cancers [40]. However, in head and neck tumors and esophageal cancers, the loss of Rab25 drives tumorigenesis [25, 41]. Additionally, in colon cancer models, Rab25 exhibits a tumor suppressive function [29]. Therefore, the ambiguous role of Rab25 in cancer initiation, progression and prognosis [42], including its role in breast cancer progression [21, 42, 43] is confusing, conflicting, and poorly understood.

In the last decade, transcriptional profiling combined with other omics technologies has reshaped our understanding of breast cancer, revising the concept of invasive ductal breast cancer as a single disease, revealing instead at least 6 intrinsic molecular subtypes with major clinical implications which were otherwise indiscernible by routine clinical parameters and pathological markers. These distinct neoplasms have unique gene signatures, cell biology, and treatment sensitivity, and hence widely differ in their outcomes. Ductal breast cancer subtypes by mRNA profiling include luminal A, luminal B, Her2 overexpressing, basal-like, claudin-low, and normal breast-like and a newly emerging reactive subtype [31, 44]. Other classification systems have split breast cancer into at least 10 subtypes [45], triple-negative breast cancer itself segregated into 6 subtypes and an additional AR (Androgen Receptor) positive variant of luminal breast cancer [46]. With further integrative analysis the classification of breast cancer is likely to become more stratified and refined. Nevertheless, it is now clear that breast cancer consists of multiple different subtypes with distinct molecular and clinical characteristics. Thus there is a pressing need to characterize markers that will enable clinical identification of the specific diseases and allocate suitable therapeutic interventions.

Considering that Rab25 is an epithelial specific GTPase [47] and is part of the 1q amplicon that is common across breast cancer subtypes [48], we hypothesized that Rab25 expression and function would be positively associated with progression of breast cancers with epithelial features. Subsequently, here we demonstrate that indeed Rab25 levels are selectively elevated in luminal B breast cancers and surprisingly suppressed in a subset of basal-like breast cancer, namely the claudin-low tumors (sometimes referred as basal B), partly due to increased genomic methylation of the Rab25 promoter region. The high Rab25 levels in luminal B tumors were associated with a significantly worsened outcome. *In vitro*, Rab25 increased proliferation, sustained indefinite growth and promoted motility in luminal B breast cancer cell lines while exerting the opposite effect on basal-like breast cancer cell lines. Further, in luminal B cells, the oncogenic effects of Rab25 were enhanced by concurrent expression of its key effector, Rab11FIP1/RCP (will be referred as RCP henceforth). Lastly, we observed that most claudin low phenotype was closely associated with a complete loss of Rab25 and was causally associated with transcription factor Snail driven Epithelial Mesenchymal Transition (EMT). Overall, in this study we discovered that Rab25 is a context dependent oncogene with a potential role in breast cancer cell plasticity (which needs further in depth analysis in the future). The goal of this study is to shed light and clarity on two distinct roles of Rab25 in cancer which could be converted as therapeutic opportunity while targeting the derailed endocytic machinery in cancers [15].

## RESULTS

### Differential expression of Rab25 within breast cancer subtypes

**Figure 1:**
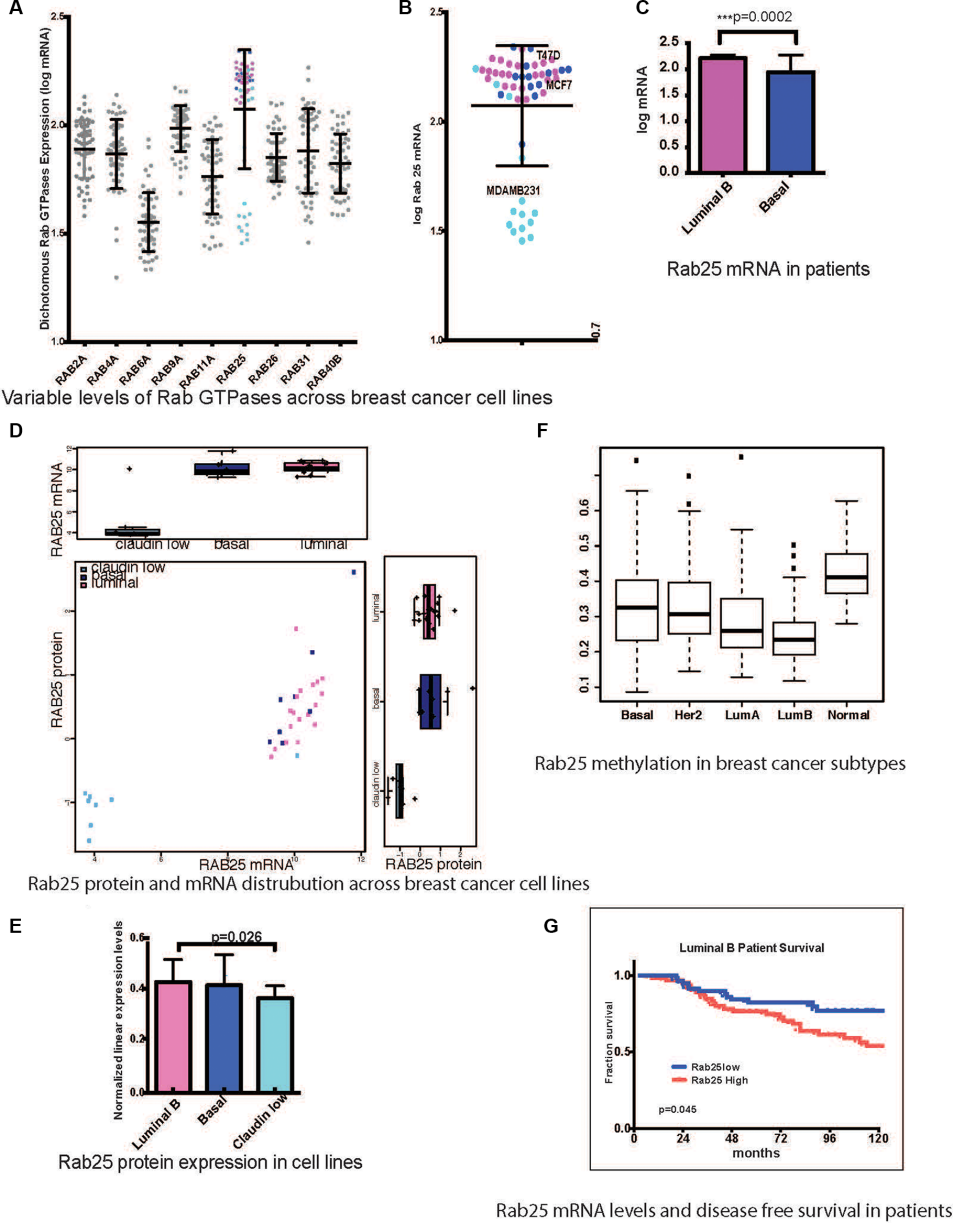
Rab25 expression is a clinical marker in breast cancer patients (**A**) mRNA levels of 9 Rab GTPases with more than 2fold range of expression across 54 breast cancer cell lines. The pink circles represent Rab25mRNA expression (log) in luminal B cell lines and light blue represents claudin low cell lines while dark blue represents basal lines. (**B**) Enlarged view of only Rab25 mRNA distribution with specific annotation of cells lines used subsequently. (**C**) Comparison of Rab25 mRNA levels between luminal B (135 tumors) and basal (71 tumors) in patients stratified based on PAM50. (**D**) Comparison of Rab25 mRNA and protein expression across breast cancer cells lines. (**E**) Graphical representation of absolute levels of Rab25 protein expression as measured by RPPA shows significant difference in between luminal B and claudin low groups. (**F**) TCGA breast cancer patient data showing different degree of methylation of Rab25 suggesting an epigenetic regulation of Rab25 gene expression. The box plot whiskers extend to 1.5 times interquartile range. The outliers in each group are shown as discrete data points. (**G**) Luminal B breast tumors are evaluated for Rab25 mRNA levels and correlated with disease free survival over 10 years (120 months). The red line shows patients with high Rab25 and blue shows patients with low Rab25.

Molecular heterogeneity in breast cancer is well defined and delineated by cell lines with distinct morphologic and molecular features. Based on transcriptional profiling these cells lines are representative of luminal B, Her2-enriched, basal and claudin-low signatures observed in patients [49, 50]. Endogenous mRNA levels of Rab GTPases 1 through 40 were analyzed across a panel of 54 breast cancer cell lines [50] in order to identify potential subtype driven aberrations in the vesicular trafficking machinery. Nine out of the 40 Rab GTPases tested had more than two-fold range of expression. Rab25 emerged as the most polarized Rab within the family with high Rab25 transcripts in luminal B lines and low Rab25 levels in claudin-low lines (Figure 1A and inset 1B). Rab25 protein levels across 54 breast cancer cell lines are shown in Figure 1E while comparison of Rab25 protein and mRNA in the same set are depicted in Figure 1D.

Comparisons of Rab25 expression in PAM50 (mRNA) classified patient cohorts (GSE2990, GSE6532, and GSE9185) reproduced a similar trend with high Rab25 mRNA in luminal B, and significantly lower levels in basal tumors (include claudin-low) (Figure 1C). This was surprising given that the 1q amplicon that contains Rab25 is prevalent across all breast cancer subtypes [51].

Subsequently, the genomic methylation of Rab25 was examined as a possible reason for Rab25 transcript suppression in basal-like and claudin-low tumors. Whole genome methylation data from The Cancer Genome Atlas project (TCGA) shows that Rab25 gene is indeed highly methylated in basal-like and normal-like tumors compared to luminal B tumors [44] (Figure 1F). Next, to evaluate the power of Rab25 as a clinical biomarker, we evaluated disease free survival of patients based on Rab25 mRNA levels. High Rab25 levels were associated with poor outcome exclusively in luminal B patients (132 patients) (Figure 1G, *p* = 0.045). Luminal A (112 patients) cases lacked a similar correlation with Rab25 expression and patient outcome (Supplementary Figure S1A, *p* = 0.267). In the basal group (65 patients), which included the claudin-low tumors, Rab25mRNA failed to predict any clear outcome (Supplementary Figure S1A, *p* = 0.134). However, the observed trend suggests that low Rab25 levels possibly associate with worse outcome (the data did not reach statistical significance, partly due to low number of claudin-low low tumors) in these patients.

### Rab25 confers growth advantage to luminal B breast cancer cell lines

To explore the context dependent role of Rab25 in breast cancer subtypes, we created stable lines with exogenously manipulated levels of Rab25 in a luminal B background (such as MCF7, T47D) as well as in basal (MCF10A) or claudin-low background (MDA231) (Supplementary Figure S1C).

**Figure 2:**
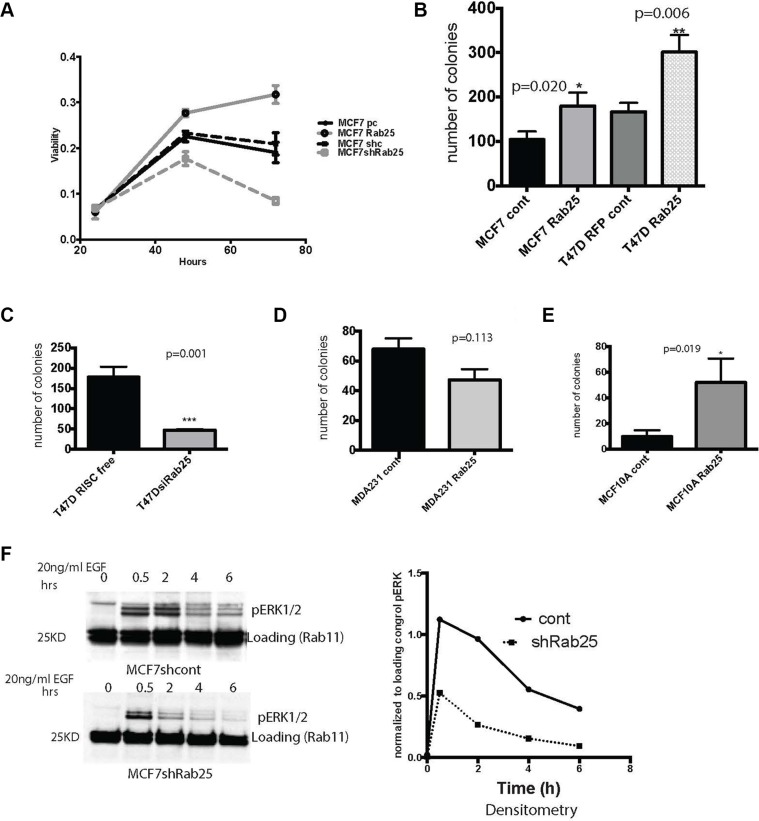
Rab25 confers growth advantage to luminal B breast cancer cells (**A**) Equal number of cells stably expressing CONTROL vectors and pcDNARab25HA or shRab25 were seeded at 50% confluence and cultured under low serum conditions for 72 hours. At each time point the cells were harvested to measure viability using MTT based assay. MCF7 pcDNA control (solid black line) and MCF7 non-silencing control (dashed black line) show baseline viability of MCF7 cells. Rab25 overexpressing cells is the solid gray line while shRab25 expressing cells shown by dashed grays line. Data represents three independent experiments. (**B**) Less than 50 luminal B cells of either MCF7 or T47D lines with stable overexpression of Rab25 were seeded in 6 well plates under low serum condition for measuring anchorage dependent growth. Plates were harvested after 10 days and number of colonies quantified. Each condition done in triplicate wells. Data represents over 4 independent experiments. (**C**) T47D cells transfected with control or siRab25 before plating in triplicate wells for anchorage dependent colony formation assay. Colonies harvested after 10 days and quantified. Data represents 3 independent experiments. (**D**) MDA231 cells with stable expression of Rab25 plated at less than 50 cells per well in 6 well plate and allowed to colonize for 10days. Data represents at least 3 independent experiments. (**E**) MCF10A cells with stable expression of Rab25 plated at less than 50 cells per well in 6 well plates and allowed to colonize for 12 days under low serum condition. Data represents at least 3 independent experiments. (**F**) Immunoblot showing MCF7 cells with endogenous Rab25 or stably expressing shRab25 that were treated with cyclohexamide and serum starved ON, followed by stimulation with 25nMol EGF for 6 hours. Top panel shows pERK signal over time in MCF7 cells expressing control shRNA and lower is data from MCF7shRab25. The densitometry representing pERK signal at each time point each time-point normalized to its loading control is shown.

Rab25 is known to recycle growth and nutrient factors in various models [11, 18, 33]. We interrogated if Rab25 confers any growth advantage, especially under limiting mitogenic conditions. When MCF7 cells with high or low levels of Rab25 were cultured in low serum (0.1%FBS) condition for three days, only cells with stable overexpression (in all cases “overexpression” represents expression of Rab25 to levels present in cells with the 1q amplicon and elevated Rab25 levels) of Rab25 remained viable while cells where endogenous Rab25 was silenced failed to thrive (Figure 2A). Notably stable overexpression of Rab11a, a close homolog of Rab25, did not confer any growth advantage under similar conditions (Supplementary Figure S2A). In MDA 231 cells with exogenous expression of Rab25, cell viability was reduced initially, but at the endpoint of the assay we did not observe a significant difference (Supplementary Figure S2B). In MCF10A cells (representing basal / basal A, cells but not claudin low) Rab25 but not Rab11a over expression, stalled viability of cells (Supplementary Figure S2C) under low serum condition. Taken together our data shows that only in luminal B breast cancers, increased levels of Rab25 facilitate cell survival.

The capacity to propel indefinite growth is a critical hallmark of cancer cells. Rab25 overexpression also significantly increased the number of colonies in luminal MCF7 (*p* = 0.02) and T47D (*p* = 0.006) cell lines compared to their controls (Figure 2B). Moreover, down-regulation of Rab25 in T47D cells markedly reduced the number of colonies (*p* = 0.001) (Figure 2C). While stable expression of Rab25 in MDA231 cells reduced the number of colonies when compared to control but the difference did not reach statistical significance (*p* = 0.113).

In MCF10A cells, exogenous Rab25 expression significantly increased the number of colonies (Figure 2E) (*p* = 0.019). Since we did not see such an outcome in the previous viability assays, we believe presence of Rab25 here probably facilitates some of the seeding mechanisms in MCF10A cells required during colony formation. In fact the MCF10A cells in the colony formation assays resembled a luminal cell line with increased Rab25 levels. Notably experiments with claudin low lines are all done using overexpression constructs because endogenous Rab25 protein is almost undetectable ruling out any gene silencing approach.

Since upregulation of mitogenic signals such as Mitogen Activated Protein Kinase (MAPK or ERK1/2) is a common feature of aggressive breast cancers [39] we tested if Rab25 levels alter the activity of ERK kinase. Wild type and shRab25 expressing MCF7 cells were treated with cyclohexamide (to inhibit synthesis of new protein), serum starved overnight and stimulated with epidermal growth factor (EGF). Levels of activated ERK measured as pERK1/2 was recorded over an extended period of time allowing the late recycling events to occur. We show here that loss of Rab25 decreased the extent and duration of MAPK signaling in the luminal cells (Figure 2F). By sustaining mitogenic signaling over a longer course of time, Rab25 mediated trafficking could provide critical survival as well as migratory advantages specifically to luminal B breast cancer cells which otherwise have limited EGF receptor expression (compared to the basal or claudin low breast cancer cells).

### Rab25 increases cell migration in luminal B cancer cell lines but inhibits migration in claudin low cell lines

The vesicular trafficking machinery is a critical regulator of cell migration since signaling modules that promote localized cytoskeletal activity such as integrins are spatially transported through the endocytic pathway to the sites of dynamic cytoskeletal activity. Not surprisingly, Rab25 is implicated in driving migratory pathways in many cancers [38, 52].

**Figure 3:**
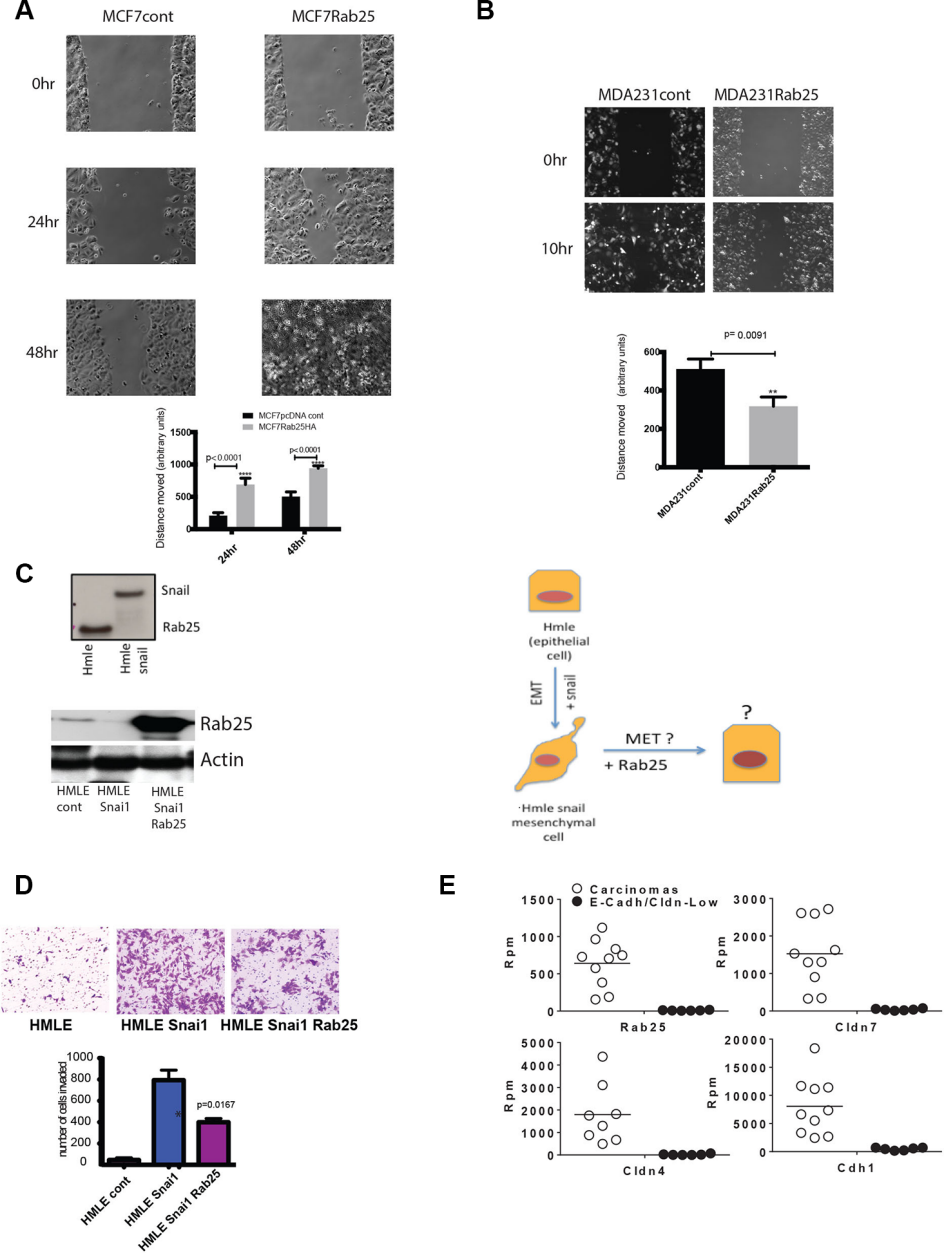
Rab25 facilitates cell migration in luminal breast cancer cells but opposes migration of claudin low cells (**A**) Shows MCF7 cells with stable Rab25 overexpression migrating to fill an artificially created scratch wound over 48 hrs. The 0 hr represents initial wounding point. The distance between cell fronts measured at time 0 is initial distance. The distance remaining after wound closure is subtracted from initial distance to calculate distance moved. Data and graph are representative of 5 independent experiments. (**B**) Shows MDA231 cells with stable Rab25 overexpression migrating to fill an artificially created scratch wound over 10 hrs. The 0hr represents initial wounding point. The distance between cell fronts measured at time 0 is initial distance. The distance remaining after wound closure is subtracted from initial distance to calculate distance moved. Data and graph are representative of 5 independent experiments. (**C**) Top panel shows immune-blot of lysates from human mammary epithelial (HMLE) cells expressing Rab25 protein that is lost with exogenous overexpression of Snail. The lower panel is an immune-blot of lysates from HMLE Snail cells where Rab25 was expressed using lenti-viral constructs. The actin panel validates equal loading. These blot are representative of multiple similar expression analysis. The side panel diagram shows HMLE as an epithelial cell before Snail is overexpressed. HMLE Snail then undergoes EMT and assumes mesenchymal morphology. With addition of exogenous Rab25 it may be possible to reverse EMT (MET) and restore epithelial features. (**D**) HMLE, HMLE Snail and HMLE Snail +Rab25 cells were assayed in a Matrigel based invasion assay using modified Boyden Chambers. Equal number of cells from each isogenic line was plated in 4 inserts. After 8 hours, the invaded cells were harvested, stained and counted. Introduction of exogenous Rab25 into mesenchymal cells (HMLE Snail) rescues cells partially from Snail-driven invasive phenotype. The data represents of 4 independent experiments. (**E**) Data from RNA Sequencing analysis of LPA mouse model showing slow growing SG Cdh1+ carcinomas in white open circles and fast growing (FGM) Cdh1-ve sarcoma-like (non epithelial) tumors. Each of the four sub-plots show that Rab25 levels correlating with Cldn7, Cldn4, and Cdh1 levels between the carcinoma and sarcoma group with non epithelial sarcoma showing undetectable levels of Rab25. Rpm stands for Reads per minute.

In MCF7 cells, overexpression of Rab25 doubled the rate of scratch wound closure, supporting that Rab25 increases collective/cooperative migration (Figure 3A, left graph, *p* < 0.0001). Notably MCF7 cells with prominent epithelial features tend to move in sheets, maintaining intact cellular junctions as indicated by claudin7 staining (Supplementary Figure S2D). Hence for these breast cancer cells, migration are typically measured in scratch wound closure assays. We also noted a slower motility index in MCF7 cells where Rab25 levels were reduced (with either shRNA or siRNA) but that data is not included here since the lengthy time frame itself added to some cell death.

In the claudin-low lines the most robust anti tumor effect of Rab25 was observed during cell migration. In the otherwise highly motile MDA 231 cells, exogenous Rab25 significantly retarded cell migration and wound closure (3B, right graph *p* = 0.0091). Notably MDA231 cells having undergone EMT [17], have lost cellular junctions and tend to migrate as single cells.

To further test cell lineage specific effects of Rab25, we used a previously described immortalized human mammary epithelial cell system (HMLE) [53] and isogenic with stable overexpression of Snail (Snai1) [54]. Molecularly, these cells are similar to basal breast cancer cells, originating from the same precursor [53]. However, with over expression of Snail, they manifest a claudin-low phenotype with upregulation of epithelial to mesenchymal (EMT) features [55, 56]. These cells with negligible expression of claudin 3, 4 or 7 and E-cadherin [49], are widely used to study EMT and known to cluster with the claudin-low patients and cell lines [55, 56]. Here we found that while parental HMLE cells (clusters with basal/basal A) have Rab25 protein expression, the introduction of Snail completely suppresses Rab25 protein (Figure 3C upper panel). The HMLE system also allowed us to address if Rab25 is important for cellular plasticity (represented diagrammatically in a third panel of Figure 3C). The parental HMLE cells being normal mammary epithelial cells are not invasive. However with the introduction of Snail, these cells undergo EMT and become highly metastatic. So we compared cell invasion in the highly metastatic HMLE Snail cells (Rab25 lost) with HMLE Snail cells expressing exogenous Rab25. We found that exogenous expression of Rab25 in the HMLE Snail cells (Figure 3C lower panel), at levels comparable to those constitutively expressed in luminal breast cancer cells, could robustly oppose Snail-driven invasion through Matrigel^TM^ (Figure 3D, *p* = 0.016). Since overexpression of Rab25 did not alter the protein levels of Snail we concluded that Rab25 does not regulate Snail expression although Snail does regulate Rab25 protein expression. The HMLE model therefore provides a potential insight as to why Rab25 (protein and mRNA) could be lost in claudin-low lines despite the presence of 1q amplicon. Snail driven gene repression (including methylation) seems a plausible explanation, which is being tested, in our ongoing studies.

Continuing our efforts to understand the dichotomy of Rab25 expression and function in the context of cell lineage we utilized an existing well-documented Lysophosphatidic Acid Receptor (LPA) driven mammary tumor model [57–59]. This model recapitulates the heterogeneity of human breast cancer in a murine context and thus represents a powerful and sensitive system to test our hypothesis [49]. Broadly the tumors in the mice were of two types. One group of tumors showed rapid growth (fast growing) while the other had significantly slower growth rate (slow growing). RNA Sequencing analysis data shows that the fast growing tumors clustered together and were enriched in EMT-like features such as loss of E-cadherin (CDH) and Claudins. Once again, these fast growing tumors with low Claudins were found to have completely lost the Rab25 transcript (Figure 3E). In contrast, the slow growing tumors, with intact E-cadherin expression had comparable (to human luminal B cancer cell lines) levels of Rab25 (Figure 3E) reinforcing a strong cell lineage specific expression and function of Rab25. This mouse model further recapitulates our observation from patients and cell lines that claudin-low signature is associated with a coordinate loss of Rab25.

### Rab25 mediates its oncogenic effects partly through the effector Rab11Fip1 (RCP) in luminal B breast cancer

The effectors recruited by activated Rab GTPases define the specificity and domain of their downstream interactions including vesicle budding, delivery, tethering. Therefore Rab and its effectors are frequently looped into positive feedback modules to ensure efficient trafficking.

**Figure 4:**
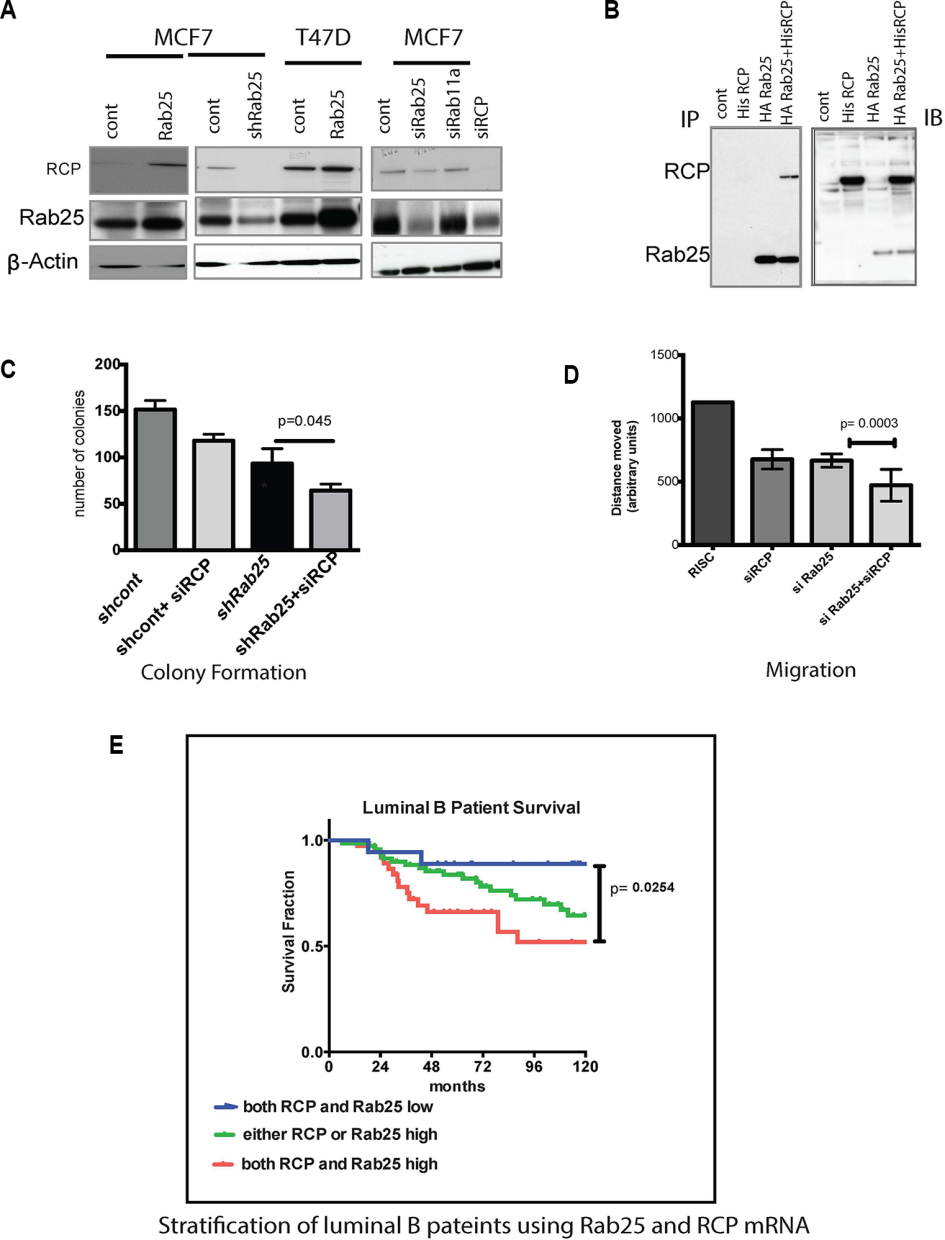
Rab25 mediates its oncogenic effects partly through RCP/Rab11Fip1 (**A**) Panel A immunoblot represents MCF7 and T47D luminal lines expressing either of Rab25 (stable), shRNARab25 (stable) or siRNA Rab25 and RCP (only in MCF7) cells. The immunoblots show levels of RCP and Rab25 in the above cell lines. Actin is used as a loading control. Data represents 2 independent experiments. (**B**) The left panel shows co-immunoprecipitaion of HA-Rab25 with His-RCP. Ovarian cancer cells (Hey) were first stably transfected with HA tagged pcDNA Rab25 and selected stable clones were then transfected with His-RCP. The His-RCP was pulled down using HA-pull-down assay and then probed for Rab25 as well as RCP. The right panel shows immunoblotting of non-immunoprecipitated lysates with anti -RCP and anti-Rab25 antibodies to validate expression of Rab25 and RCP. Data represents of 5 independent experiments.(**C**) Shows an anchorage dependent colony formation with a combined loss of RCP and Rab25 in MCF7 cells. The MCF7 cells in which RCP is silenced are compared with MCF7shRab25 stable cells also with silenced RCP. Following siRCP transfection less than 50 cells were plated in 6 well plates and colonies populated for 10 days before were being harvested and quantified. Data shown is *n* = 3. (**D**) In scratch wound closure assays using T47D cells, loss of endogenous Rab25 and RCP by introduction of siRab25 and siRCP has additional impact on retarding cell migration compared to the individual genes. T47D cells were subjected to siRNA transfection 24hr before they were plated for the wound assay in triplicate. Wound healing time was limited to 24 hr from the time of initial scratching. Data shown is *n* = 3. (**E**) Rab25 and RCP gene expression when combined can better risk stratify luminal B breast cancer patients. Data with 135 breast cancer patients categorized by PAM50 as luminal B shows that the 10-year survival proportion of patients with both high Rab25 and high RCP (red line) is the worst outcome in terms of disease free survival. The blue line marking patients with both low Rab25 and low RCP shows the best prognosis. The green line representing patients with either Rab25 or RCP is high shows better prognosis than those with both high. However these patients fare poorly compared to cases where Rab25 and RCP are both low.

The Rab11FIPs are the main effectors of Rab 11 family members including Rab25 [60]. Additionally, Rab11Fip1/RCP is an oncogene in breast cancer, triggering activation of the Ras-MAPK pathway [60]. Next, to confirm if Rab25 mediated its oncogenic effects through RCP in luminal B breast cancer, we altered levels of Rab25 or RCP and interrogated the cells for expression as well as functional assays. We found that in MCF7 and T47D cells, Rab25 and RCP expression levels were coordinately regulated, consistent with a positive feedback loop or the presence of a protein complex necessary for the stability of the heterodimer (Figure 4A). We validated the physical interaction between Rab25 and RCP by co-immunoprecipitating stably expressing HA-Rab25 along with His-RCP in multiple lines (MCF7, T47D, MDA 231, Hey) of which representative data from ovarian Hey cancer cells are shown here (Figure 4B). Subsequently utilizing Rab25 sensitive assays including colony formation (Figure 4C) and migration (Figure 4D), we examined the functional relevance of Rab25-RCP interactions. In agreement with our previous findings, loss of Rab25 reduced growth and migration of the luminal B lines, as did loss of RCP independently (Figure 4C and 4D). Additionally, when both Rab25 and RCP levels were suppressed, the ability to form colonies (Figure 4C, *p* = 0.045) as well as migration potential were further compromised supporting that in luminal B cells Rab25 and RCP work in tandem.

Existing literature supported an oncogenic role for RCP in both luminal and basal breast cancers. But new evidence highlights a tumor suppressor role in ERB2 driven breast cancers [61]. This led us to opt for a coordinate analysis of RCP and Rab25 within the luminal B patient subgroup to evaluate if our *in vitro* observations translate in patients. In the patient data we sampled, RCP expression alone was not a predictor of outcome in luminal B (Supplementary Figure S3B) or basal (Supplementary Figure S3C) patients although RCP transcript level was variable between luminal A, luminal B and basal tumors (Supplementary Figure S3C). Notably, in this data set as well as other tumor databases, Rab25 and RCP expression are not always highly correlated (Supplementary Figure S3D).

However, within the luminal B breast subtype, incorporating RCP mRNA expression along with Rab25 provided a finer stratification leading to better prediction of outcome. Patients with high Rab25 and high RCP mRNA expression fared the worst compared to those where either one was high (Figure 4E, *p* = 0.0254). As expected, the best prognosis for luminal B breast cancer subtype occurred in patients with both low Rab25 and low RCP levels. It is important to identify combined roles of Rab25 and RCP in the luminal B system to better determine targeted therapeutics. For example, our lab and others have shown than Rab 25 increases PI3K, EGFR and integrin signaling. Combining the pathways altered by Rab25 with those altered by RCP (such as Ras/ MAPK pathway) implies that co-targeting of EGFR and PI3K/AKT pathways in luminal B patients with elevated RCP and Rab25 could have clinical benefits.

## DISCUSSION

Cancer is a disease of extraordinary complexities. Disparate cancers share fundamental qualities while cancer within the same organ often produce distinctly different diseases. In the domain of personalized cancer therapeutics, a finer stratification of patients within each breast cancer subtype is expected to increase therapeutic efficiency and improve the quality of life of the patient. Rab25 and RCP may act as biomarkers to further stratify breast cancer patients further.

The vesicular trafficking machinery plays a major role in normal cellular physiology to regulate the activity and fate of mitogenic proteins as well as proteins that allow the cell to interact with each other and the ECM. Thus, in theory, the endocytic machinery is powerful tool to regulate protein function and cancer cells appear to coopt this function by derailing key mediators of the trafficking machinery. Here we have identified the Rab25 vesicular trafficking protein as a biomarker of disease progression in the luminal B subtype of breast cancer. Additionally, we found that absence of Rab25 is a clinically reliable tool to detect claudin-low breast cancers.

In this study we have illustrated a context dependent role for Rab25, where it acts as an oncogene in luminal B breast cancer and as a tumor suppressor in claudin-low tumors. The nature of this molecule and its pleiotropic effects on multiple aspect of cellular function make it rather challenging to fully elucidate the underlying mechanisms. However, others and us have shown that the most prominent biological effect of derailed Rab25 mediated trafficking is altered cell migration [11, 24]. This process is aberrant in basal-like tumors that have already undergone EMT potentially making Rab25 expression deleterious to the rate of migration. Strikingly, Rab25 expression was not only decreased as a consequence of enforced expression of Snail, re-expression of Rab25 was sufficient to essentially reverse the effects of Snail on cell migration. In our studies exogenous introduction of Rab25 did not alter E-cadherin or Claudins (3,4,7) protein levels in MDA231 cells (data not presented), however Rab25 retains the potential to alter localization of other epithelial junction proteins. Thus while Rab25 is a key player in the EMT phenotype in claudin low breast cancer the exact mechanism remains unknown with the potential that the function of proteins mediating apicobasal polarity as well as other transcriptional pathways remains to be explored. In contrast, in relatively non-migratory luminal B cells, Rab25 may increase the migration rate. Further, our study cautions against generalization of functions because Rab25 drives cell migration in luminal B tumors while it opposes EMT and mobility in claudin-low tumors. The opposite effects may be due to different modes of motility in luminal B tumors that are relatively epithelial and polarized moving as sheets compared to claudin low tumors cells that have undergone EMT and tend to migrate as single cells. Currently our ongoing research needs to be done to systematically map out the role of Snail in transcriptional regulation of Rab25 including its role in methylation of the Rab25 gene. Notably, Snail transcription factor itself is expressed in both MCF7 (less) and MDA231 cells. Others have reported that overexpression of WT Snail in MCF7 does not lead to EMT, but expression of a mutant form of Snail 6SA which was less degradable allowed the MCF7 cells to lose E-cadherin and undergo a mesenchymal transformation [62].

The list of effectors of Rab25 are increasing and while Rab11Fip1 or RCP was found to be a functional accomplice of Rab25 in luminal B cancers and is key for better stratification of luminal B patients, we are yet to understand the dynamics of Rab25-RCP in the other breast cancer subtypes. At this time, our findings aid in better stratification of luminal B patients using the Rab25-RCP axis. Since Rab25 is known to recycle integrins as well as growth factors, we speculate that high levels of Rab25-RCP expression may identify a subset of luminal B patients who may benefit from anti EGFR therapy or anti-integrin therapy.

Finally, we found a causal association of Rab25 with cellular lineage in the context of breast cancer that needs further evaluation. Why Rab25 is methylated in basal cells and present at low levels is basal and claudin-low cells are being currently investigated in our lab. In multiple models we have found that the transcription factor Snail can completely abrogate Rab25 protein levels. However, sensitivity of Rab25 expression to Snail is also dependent on the cellular lineage. It would be intriguing to associate a vesicular trafficking protein like Rab25 with stem cell like properties of breast cancer cells. Consistent with this contention, recent studies [40] [63] suggest that the vesicular trafficking machinery plays a key role in maintaining pleuripotency. Overall, our previous work has shown that in luminal cells Rab25 recycles cell cycle proteins such as EGFR [17] very efficiently leading to enhanced pro-tumorigenic cell signaling. However in claudin low cells, Rab25 appears to trap EGFR in the cytosol (unpublished data). Thus a Rab25 may target cargo to different subcellular compartments in different breast cancer subsets thus contributing to the differential function in these subsets.

## MATERIALS AND METHODS

### Cell lines and constructs

Breast cancer cell lines MCF7, T47D, MDA231, MCF10A were originally obtained from ATCC and cultured in RPMI 1640 with 5% FBS. Cell lines were transduced by lentiviral Rab25 constructs (Open Biosystem, Dharmacon, Lafayette, CO) followed by Blasticidin selection (30 μg/ml) up to 7 days. Subsequently limited serial dilution was performed and single cells were allowed to expand to separate colonies for genetically homogenous population. Two independent clones were obtained for each constructs and target gene expression was evaluated with western blotting. Additionally, a sequence confirmed hemagglutinin epitope-tagged *RAB25* (Invitrogen, Carlsbad, CA) expression vector on pcDNA 3.1 backbone previously constructed by PCR amplification as described previously [32] was used in specific assays.

To generate Rab25 knockdown both shRNA (Arrest GIPZ lentiviral shRNA particles from Open Biosystems, ThermoFisher Scientific Inc) and siRNA Rab25 (On-Target Plus Smartpool^TM^ small interfering RNAs Dharmacon, Lafayette, CO) were used following manufacturer's instructions. The siRNA constructs were introduced into the cells using Lipofectamine RNAi MAX (Invitrogen, Carlsbad, CA) according to the manufacturer's instructions. A scramble RNAi negative control was purchased from Ambion. Protein levels were evaluated by Western blotting at 72 hr post transfection. shRab25 infected cells were selected and routinely cultured with 1 μg/mL puromycin (Sigma-Aldrich). The identities of all cell lines were verified using AmpF/STR Identifier kit (Applied Biosystems). The His-RCP construct was a kind gift from Dr. James Norman (Beatson Institute Glasgow, UK). Immortalized human mammary epithelial cells (HMLE), a gift from Sendurai Mani (M.D. Anderson Houston,Tx) were maintained as previously described [53]. Cell lines were routinely tested for mycoplasma infection using a MycoTect kit (Invitrogen).

### Proliferation assay

Breast cancer cells stably expressing Rab25 or shRab25 were seeded in triplicate in 96 -well plates in growth medium. The next day the wells were washed twice with sterile PBS and fresh media added with 0.1% FBS. Cell proliferation was assessed at 24, 48, and 72 hours by a 2 hr incubation with 3-(4,5-dimethylthiazol-2-yl)-2,5-diphenyltetrazolium bromide (MTT), followed by lysis in acidic isopropanol (0.35% HCl in isopropanol) and measurement of absorbance at 570 nm.

### Colony formation assay

For anchorage-dependent colony formation, stable lines with Rab25 overexpression or knockdown were seeded at < 50 cells per well in 6 well plates in 1% FBS containing media and monitored for 10–12 days. Additionally, for T47D breast cancer lines, 1 × 10^6^ cells were transfected with either control siRNA or si*RAB25* and 48 hours posttransfection, cells were trypsinized and replated in to six-wells plates at < 50 cells per plate and monitored for 10–12 days. Cells were fixed with 4% PFA and stained with 0.5% Crystal Violet. The number of colonies formed was counted and graphically represented.

### Migration assay

Cell migration was measured using a modified Boyden chamber (BD Biosciences). 10^5^ cells were placed in the upper compartment of the Transwell chamber. Lower chambers were filled with 1% FBS-containing medium. Cells were allowed to migrate for 8 h. Migrated cells were fixed and stained with 0.5% crystal violet. Four randomly chosen fields were quantified at 10× objective.

### Wound closure assay

About 6000–8000 cells were plated on either side of the silicon insert in the Ibidi wound assays chambers (silicon insert provides a defined cell-free gap, Cat # 80206 Ibidi, LLC Verona, WI) in 80 ul of growth medium. The cells were allowed to form a confluent monolayer overnight. The following day the silicon insert was gently removed with sterile forceps to expose the cell- free gap. The plate was rinsed gently with sterile PBS to remove any dislodged cells and replaced with 1% FBS containing medium and incubated till the cell-gap wound is closed. Images were recorded at 5 different coordinates along the wound at time zero (wound created) till the wound is closed in any single condition tested. The distance migrated by the cells to close the wound is calculated by subtracting the final cell free gap from the initial cell free gap at reach coordinate.

### Antibodies, Western blotting and immunofluorescence

Whole-cell lysates for western blotting were extracted with RIPA (25 mM Tris-HCl pH 7.6, 150 mM NaCl, 1% NP-40, 1% sodium deoxycholate, 0.1% SDS, protease, and phosphatase inhibitor cocktail). Cell lysates (30 μg) were loaded onto SDS-PAGE and transferred to a PVDF membrane. Protein expression was detected with an enhanced chemiluminescence western blot detection kit (Amersham Biosciences). The blots were then probed with various antibodies, such as anti-pMAPK (Cell Signaling), anti pAKT (Cell Signaling), Anti Snai-1 (Cell Signaling) anti-ERK2 (Santacruz), anti-Caludin 7 (Neomaker).

Protein quantification of whole-cell lysates and Western blotting using primary antibodies for Rab25 (Cell Signaling 4314; 1:500) and RCP (Gift from Dr Jim Norman1:1000) and secondary antibodies, antirabbit or antimouse immunoglobulin G (IgG) horseradish peroxidase–linked secondary antibody (Cell Signaling Technology; 1:2000). Whole-cell lysates for immunoprecipitation were prepared using lysis buffer provided in Sigma HA-Immunoprecipitation Kit (IP0010-1KT Sigma Aldrich, St. Louis, MO), and protease inhibitors. The lysates were precleared by incubating with 1:1 slurry of protein A/G agarose (Santa Cruz Biotechnology) for 1 hr at 4°C, and then immunoprecipitated with antibodies against HA overnight at 4°C. The immune complexes were collected by incubation with protein A/G agarose for 4 hr before being resolved by SDS-PAGE. Normal IgG was used as negative control. For immunofluorescence, cells were fixed using acetone methanol or 4% PFA and stained following methods detailed in [64].

### RPPA and TCGA analysis

Protein expression data were collected by RPPA technology (Carey et al. 2010). The RPPA's spot signal intensity data were processed by the R package Super-Curve available at http://bioinformatics.mdanderson.org/OOMPA, which generates the relative log2 expression value for each protein. The log2 expression data were then corrected by median-centering protein expression across the samples and then across antibodies. After data correction, a two-way unsupervised hierarchical clustering heat-map was generated using the Next Generation Clustered Heat Map (NGCHM) tool. For correlation matrix analysis, Pearson correlation coefficient was used to measure correlation strength between expression profiles of protein pairs across all samples, and Ward was used as linkage rule to evaluate the distances between clusters. The heatmap color legend is a histogram that displays the ranges of the relative log2 protein expression levels. Proteins with a *p*-value < 0.0001 were considered highly correlated.

### Rna seq

The RNA Seq data was run in the MDACC CCSG core and details were published elsewhere [58]. To characterize transcriptional landscapes of the Mouse Derived Syngeneic transplants (MDST), RNAseq analysis was performed on a representative group of five Slow Growing (SG)-CDH1+ carcinomas, one Fast Growing (FG)-mixed squamous carcinoma, and three Fast Growing Mixed (FGM)-like sarcomas. The SG-CDH1+ MDST were transcriptionally heterogeneous as they clustered in six separate branches and conversely, all FGM-like MDST grouped in a single cluster indicating greater homogeneity.

### Statistical analysis

All experiments were independently repeated at least twice. Data of the *in vitro* assays were derived from triplicates of three independent experiments and are presented as means ± SD. When two groups were compared, the Student *t*-test was used (*P* < 0.05 was considered significant).

To estimate the clinical relevance of Rab25 expression in breast cancer subtypes, patient data on breast tumors from publicly available sets were used (GSE2990, GSE6532, and GSE9185). These sets are from the same institute and same platform (Affy U133) and were normalized together, and the median was centered before further analysis. Subtypes were stratified based on gene signature [65, 66]. Patients with no further follow-up information are represented by a vertical tick at last point of contact and are weighted in the Kaplan-Meier analysis. The median centered date for each gene of interest were sorted to identify high (positive) expression or low (negative expression) prior to calculating patient survival. To identify the dichotomous of Rab GTPases in breast cancer a publically available mRNA microarray data from a panel of 54 breast cancer cell lines were used [67]. The mRNA and protein expression differences between groups of breast cancers and cell lines were assessed using *t* tests and one-way ANOVA. Statistical analyses were conducted using Prism 5.0c (GraphPad Software) *P* ≤ 0.05 (2-sided) was considered significant.

## SUPPLEMENTARY MATERIALS FIGURES


