# Supplementary material for: Rab25 acts as an oncogene in luminal B breast cancer and is causally associated with Snail driven EMT
Source: Oncotarget. 2016 May 31;7(26):40252–65. doi: 10.18632/oncotarget.9730 (PMC5130006; doi:10.18632/oncotarget.9730)
Supplement: Supplementary file 1 [file oncotarget-07-40252-s001.pdf]

Rab25 acts as an oncogene in luminal B breast cancer and is causally associated with Snail driven EMT

Supplementary Materials

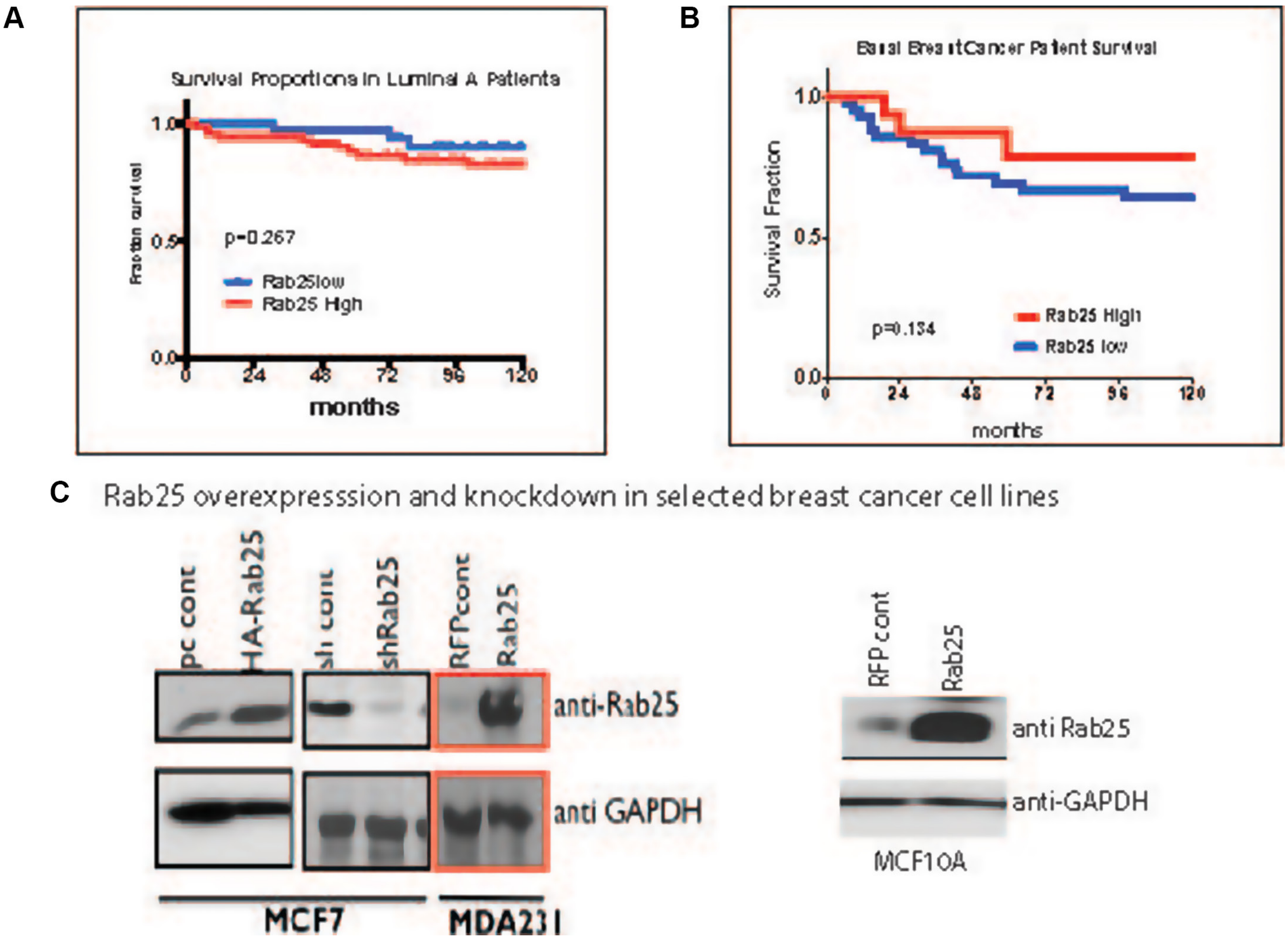

Supplementary Figure S1: Rab25 levels in patients and cell lines. (**A**) The left panel shows Kaplan Meyer curves with survival proportion over a10 year span based on Rab25 mRNA levels in 112 patients with luminal A breast cancer. The red line represent tumors with high Rab25 and the blue line represent those with low Rab25. (**B**) The right panel shows Kaplan Meyer curves with survival proportion over a 10-year span based on Rab25 mRNA levels in 65 patients with basal breast cancer. The red line represents tumors with high Rab25 and blue line represents those with low Rab25. (**C**) The left panel shows endogenous levels of Rab25 in luminal B cell line namely MCF7, which was enhanced with exogenous HA-Rab25 expression (stable). Endogenous level of Rab25 was markedly diminished with stable expression of small hairpin against Rab25 (mid panel). The next panel shows claudin low breast cancer line MDA231 have no detectable intrinsic Rab25. Stable expression of extrinsic Rab25 leads to robust Rab25 protein expression as detected by immune blotting. The far right panel shows endogenous and overexpression in MCF10A (basal) cells. GAPDH was used as a loading control for all the assays. Data represents at least 4 independent experiments.

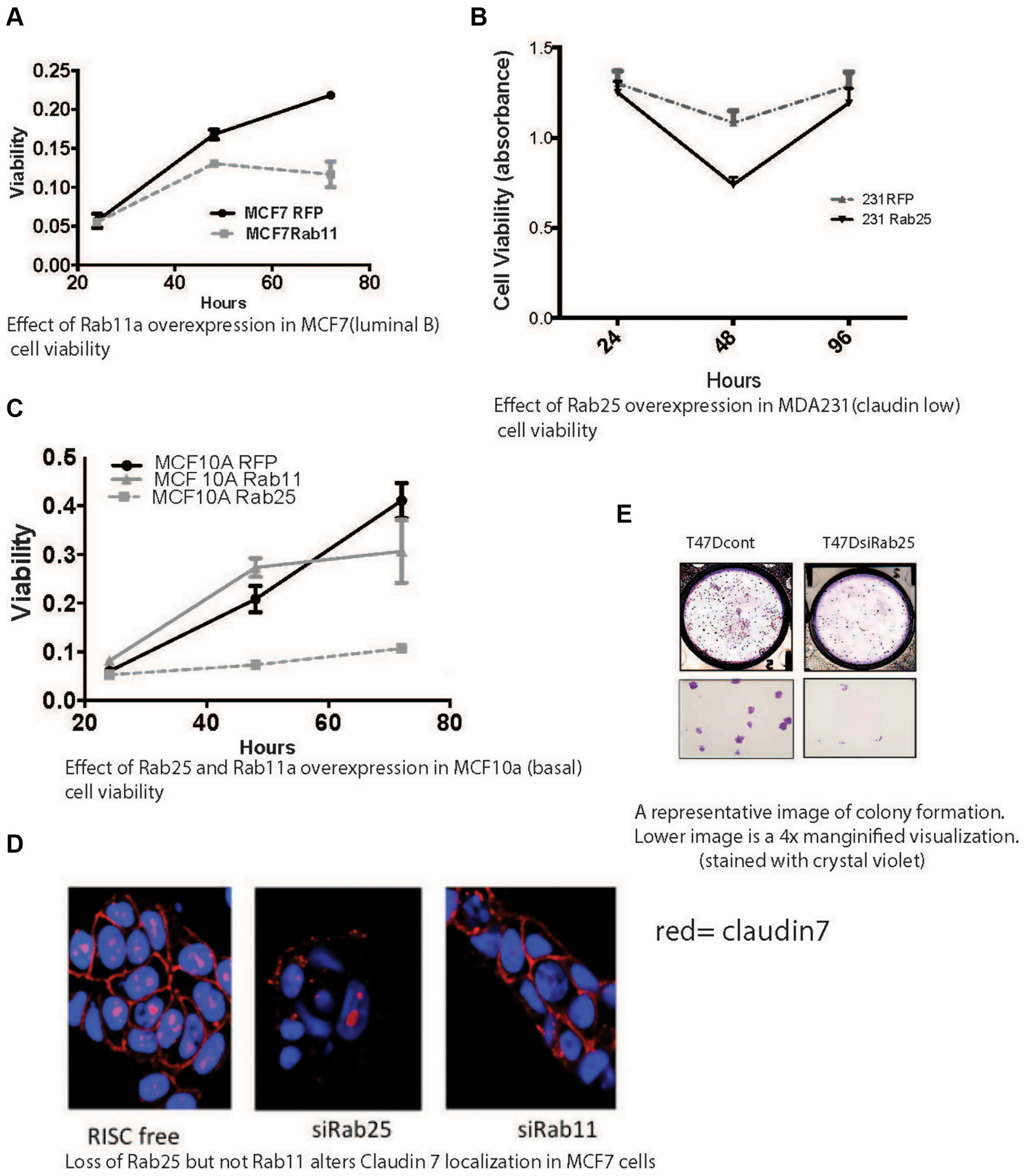

Supplementary Figure S2: Rab25 or Rab11 dependent cell viability in breast cancer cell lines. (**A**) Equal number of MCF7 cells stably expressing Rab25 or Rab11 were seeded at 50% confluence and cultured under low serum conditions for 72 hours. At each time point the cells were harvested to measure viability using MTT based assay. MCF7 control (solid black line) shows baseline viability of MCF7 cells. Rab11overexpressing cells are the dashed gray line. Data represents 2 independent experiments. (**B**) Equal number of MDA231 cells stably expressing Rab25were seeded at 50% confluency and cultured under low serum conditions for 72 hours. At each time point the cells were harvested to measure viability using MTT based assay. MDA231 control (dashed gray line) shows baseline viability of MDA231 cells under experimental conditions. The solid black line represents Rab 25 overexpressing cells. Data represents 3 independent experiments. (**C**) Equal number of MCF10A cells stably expressing Rab25 or Rab11 were seeded at 50% confluence and cultured under low serum conditions for 72 hours. At each time point the cells were harvested to measure viability using MTT based assay. MCF10A control (solid black line) shows baseline viability of MCF10A cells under experimental conditions. Rab25 overexpressing cells is the dashed gray line while Rab11a expressing cells are the solid gray line. Data represents 2 independent experiments. (**D**) Shows confocal images of MCF7 cells treated with siRab25 or siRab11 before fixing and staining for tight junction protein claudin 7(red). The cell nucleus is stained with DAPI. Image is representation of multiple fields and 3 independent experiments. (**E**) Shows representative wells from colony formation assay with T47D control and T47D expressing siRab25 lines. The colonies were stained with crystal violet solution for visualization.

A RCP mRNALevels in breast cancer patient subtypes

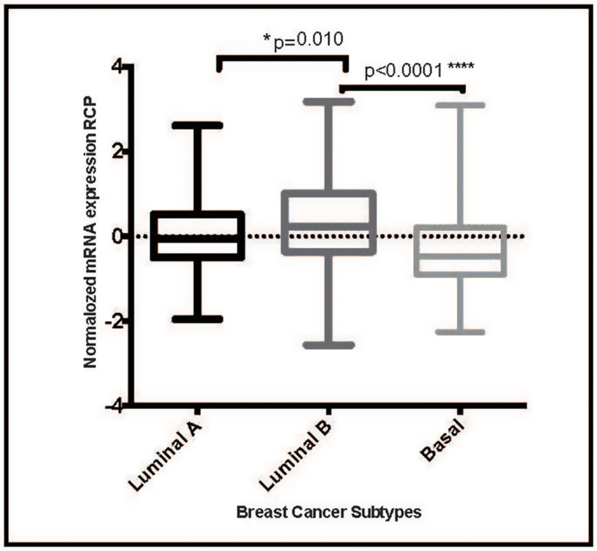

S3B and C RCP levels and disease free survival in luminal B and Basal patients

B

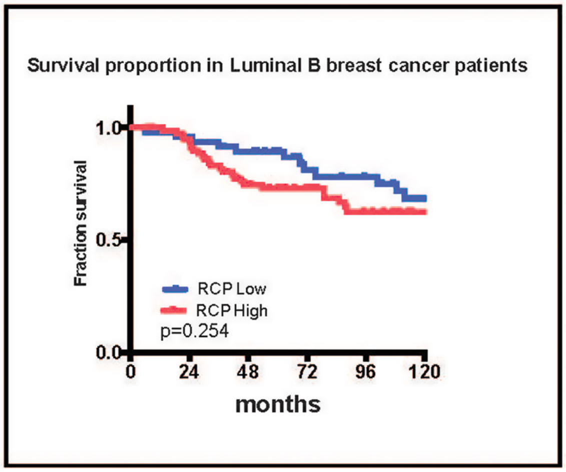

C

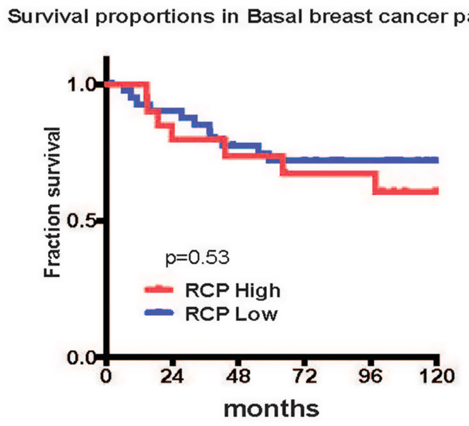

D Rab25 and RCP correlation in patients

Luminal B cohort

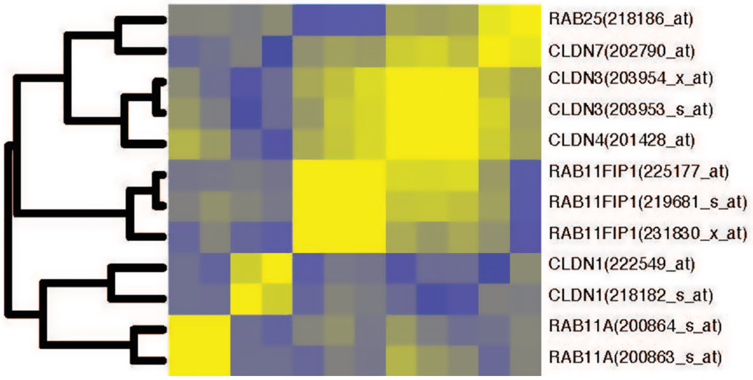

Supplementary Figure S3: RCP levels in breast cancer patients and prognosis. (**A**) Shows RCP expression across breast cancer subtype including luminal A, luminal B and basal tumors from patients. Luminal B tumors have higher expression of RCP than both their luminal A counterparts as well as basal tumors. (**B**) The left panel shows Kaplan Meyer curves with survival proportion over a10 year span based on RCP mRNA levels in 132 patients with luminal B breast cancer. The red line represent tumors with high RCP and the blue line represent those with low RCP mRNA. (**C**) The right panel shows Kaplan Meyer curves with survival proportion over a 10-year span based on RCP mRNA levels in 65 patients with basal breast cancer. The red line represents tumors with high RCP and blue line represents those with low RCP. (**D**) Heatmap depicting the correlation of Rab25 mRNA with Claudins 1,3,4 and 7), RCP (Rab11Fip1) and Rab11a mRNA in luminal B patients (cohort = 132 patients).
